# Structural, Morphological, and Electron Transport Studies of Annealing Dependent In_2_O_3_ Dye-Sensitized Solar Cell

**DOI:** 10.1155/2015/403848

**Published:** 2015-06-04

**Authors:** S. Mahalingam, H. Abdullah, S. Shaari, A. Muchtar, I. Asshari

**Affiliations:** ^1^Department of Electrical, Electronic and System Engineering, Faculty of Engineering and Built Environment, Universiti Kebangsaan Malaysia, 43600 Bangi, Selangor, Malaysia; ^2^Institute of Microengineering and Nanoelectrics, Universiti Kebangsaan Malaysia, 43600 Bangi, Selangor, Malaysia; ^3^Department of Mechanical and Materials Engineering, Faculty of Engineering and Built Environment, Universiti Kebangsaan Malaysia, 43600 Bangi, Selangor, Malaysia; ^4^Unit Pengajian Asas Kejuruteraan (UPAK), Faculty of Engineering and Built Environment, Universiti Kebangsaan Malaysia, 43600 Bangi, Selangor, Malaysia

## Abstract

Indium oxide (In_2_O_3_) thin films annealed at various annealing temperatures were prepared by using spin-coating method for dye-sensitized solar cells (DSSCs). The objective of this research is to enhance the photovoltaic conversion efficiency in In_2_O_3_ thin films by finding the optimum annealing temperature and also to study the reason for high and low performance in the annealed In_2_O_3_ thin films. The structural and morphological characteristics of In_2_O_3_ thin films were studied via XRD patterns, atomic force microscopy (AFM), field-emission scanning electron microscopy (FESEM), EDX sampling, and transmission electron microscopy (TEM). The annealing treatment modified the nanostructures of the In_2_O_3_ thin films viewed through FESEM images. The In_2_O_3_-450°C-based DSSC exhibited better photovoltaic performance than the other annealed thin films of 1.54%. The electron properties were studied by electrochemical impedance spectroscopy (EIS) unit. The In_2_O_3_-450°C thin films provide larger diffusion rate, low recombination effect, and longer electron lifetime, thus enhancing the performance of DSSC.

## 1. Introduction

A commercially viable dye-sensitized solar cell (DSSC) through low-cost processes for electricity generation that exhibits realistic energy-conversion efficiency was first reported by O'Regan and Graetzel in 1991 [1]. In common, DSSCs are composed of a metal oxide semiconductor as a photoanode, organic dyes, electrolyte, and platinum counter electrode [2]. The advantages of DSSCs are low cost, flexibility, being environmental friendly, and fabrication ease that make them be more favorable than conventional silicon solar cells [3]. Furthermore, the performance of the highest reported efficiency (~15%) was achieved by TiO_2_-based DSSC [4].

However, the evolution of favorable semiconductor materials used as photoanode in DSSCs that show upgraded solar cell performance is still essential. Some photoanodes with higher electron mobility improve the electron transport throughout the semiconductor layer [5]. Many developments have been going on in the recent years in other semiconductor materials for DSSCs, such as ZnO [5], SnO_2_ [6], and In_2_O_3_ [7].

Research on In_2_O_3_ is very rare in photovoltaic materials due to its weak photoelectroactivity and poor charge carrier transport in In_2_O_3_ [8], thus decreasing the power conversion efficiency of the cell. For example, the previous research on In_2_O_3_ as photovoltaic material exhibited a low current density of 0.75 mA/cm^2^ [9] and 3.83 mA/cm^2^ [8] which is remarkably low compared to other metal oxides such as TiO_2_ (9.49 mA/cm^2^) [10] and ZnO (6.1 mA/cm^2^) [11]. In spite of that, In_2_O_3_ has been used as a dopant in TiO_2_ to enhance the device performance where it helps to increase the open-circuit voltage (*V*_oc_) by elevating the energy level of the oxide [12].

In this research, In_2_O_3_ nanoporous enhanced by annealing treatment, believed to act as a direct transport layer for photogenerated electrons leading to low recombination rate and longer electron life time in DSSCs, were prepared by spin-coating technique [13]. In addition, the porous morphology of In_2_O_3_ has greater surface area which makes it easier for dye loading and increases the overall power conversion efficiency [9]. The crucial aim of the paper is to fabricate and characterize In_2_O_3_ cell by varying the annealing temperatures in the range of 350°C, 450°C, and 550°C as a photoanode in DSSCs in the view point of material properties to get a higher photovoltaic efficiency. The effects of annealing temperatures on the thin films were studied. This work compares the structure, morphology, photovoltaic properties, and electron transport properties through out the cell. The method used to develop the DSSC is simple, inexpensive, and not harmful to the environment.

## 2. Experimental Details

### 2.1. Preparation of In_2_O_3_ Thin Film

**Figure 1:**
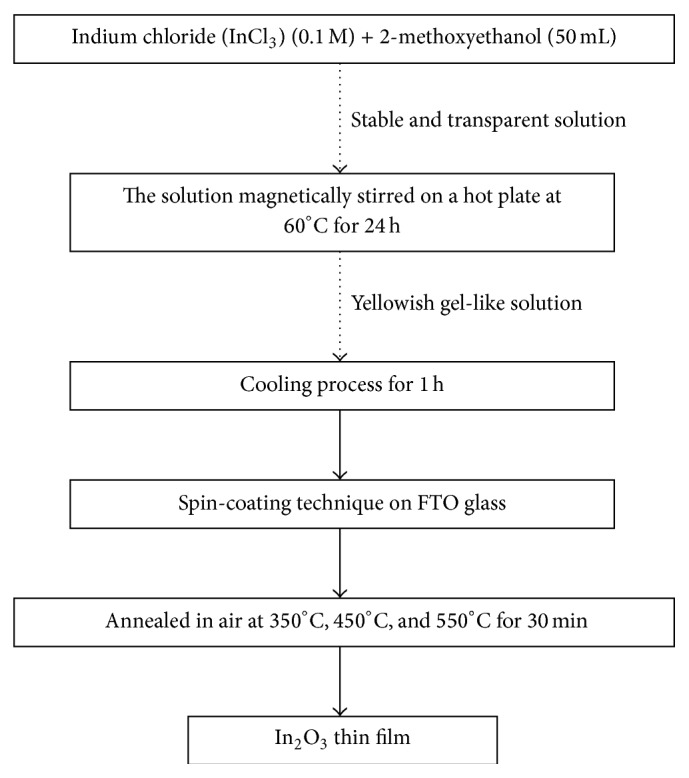
Flow chart of the In_2_O_3_ thin films preparation process.

In_2_O_3_ thin films were prepared by using sol-gel method via spin-coating technique as in Figure 1. A stable transparent aqueous solution was formed by adding 0.1 M of indium chloride (InCl_3_) into 50 mL of 2-methoxyethanol. The solution was stirred on a hot plate at 60°C for 24 h to form a yellowish gel-look solution. The heated solution was kept for cooling for 1 h before it can be deposited on a clean fluorine-doped tin oxide (FTO) coated glass substrates. The gel-look solution was spun in a spin coater (Model WS-400BX-6NPP/LITE) by coating five layers on the conducting substrates. The coated substrates were heated at a 10-minute interval before starting a new layer. The obtained In_2_O_3_ thin films were annealed at different temperatures of 350°C, 450°C, and 550°C for 30 min in air. The samples were taken out from the furnace when the temperature dropped to 40°C and cooled down to room temperature.

### 2.2. Fabrication of DSSC

The dye immersion process is the first step in fabricating the DSSC. Ethanolic N719 dye (0.5 mM) was used to immerse the annealed In_2_O_3_ thin films. The thin films were immersed in the dye for 1 day in a glass petri dish. The immersed thin films were rinsed in ethanol to remove excess dye from the FTO substrate. Consequently, the counter electrode (CE) was prepared by depositing platinum paste a clean FTO glass substrate by screen printing technique. The platinized FTO is then annealed in air at 400°C for 1 h. The DSSC was fabricated by assembling the annealed CE and immersed photoanode by sandwiching them together. A parafilm layer and two binder clips were used to fix the cell. Finally, the electrolyte (Idolyte MPN 100 Solaronix SA) was injected into the cell. The active area of the resulting DSSC is 1 cm^2^.

### 2.3. Characterization of the Thin Films

X-ray diffractometer (XRD) (Siemens D-5000) confirmed the orientation and crystallinity of In_2_O_3_ nanostructures. The surface roughness of the thin films was analyzed by atomic force microscopy (AFM) (NTEGRA Prima). Moreover, the field-emission scanning electron microscope (FESEM) (Zeisz Supra) packed along with energy dispersive X-ray (EDX) unit characterizes morphology and structural properties of the thin films. A transmission electron microscopy (TEM) (Philips, CM21, 20–200 kV) was used to observe the internal structure of the thin films. The photovoltaic performance of the annealed thin films was evaluated through photocurrent density-voltage (*J*-*V*) curve measurement via linear sweep voltammetry unit (Gamry Physical Electrochemistry (PHE200)) under 1000 W/m^2^ illumination (1.5 AM) of OSRAM halogen lamp, 50 W. The electron movement throughout the DSSC was investigated by an electrochemical impedance spectroscopy (EIS) unit (Gamry Series G300 Potentiostat).

## 3. Results and Discussions

### 3.1. Structural Characterization

**Figure 2:**
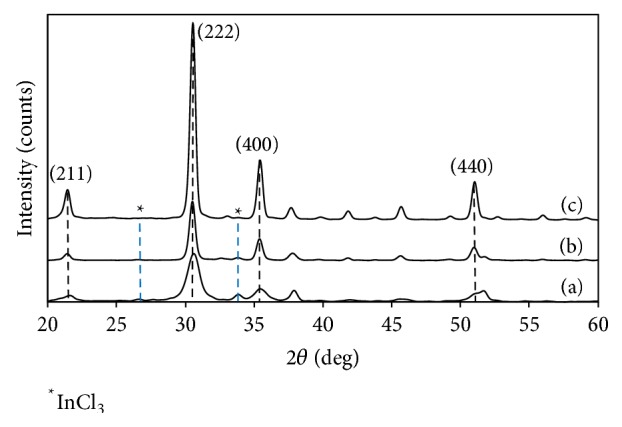
XRD spectrum of the In_2_O_3_ thin films annealed at 350°C, 450°C, and 550°C.

The XRD analysis was carried out with 2*θ* range of 20° to 60°.  Figure 2 shows the XRD patterns of the annealed In_2_O_3_ thin films. The XRD reflections confirm the cubic phase of In_2_O_3_ with bravais lattice structure of body-centered cubic, space group of *Ia*-3, number 206, and *Z* = 16. The lattice constant of *a* is 10.117 Å for the bulk In_2_O_3_ crystal with cubic structure (JCPDS number 01-071-2194). The diffraction peaks of In_2_O_3_ were identified at 2*θ* = 21.504°, 30.575°, 35.475°, and 51.043°, corresponding to the (*h* *k* *l*) miller indices of (211), (222), (400), and (440). A preferential orientation along *a*- was traced at plane (222). The peaks attributing to InCl_3_ (JCPDS number 00-034-1145) completely disappeared at high annealing temperatures and thus all the diffraction peaks are due to cubic In_2_O_3_ phase. The effect annealing temperature towards the phase transformation from InCl_3_ to In_2_O_3_ can be clearly observed through XRD analysis. The orientation of (111) crystal plane is predominant as the annealing temperature was increased. The crystallinity of the thin films was enhanced as the annealing temperature was increased based on the gradual increase in the intensity of the peaks as observed in the XRD spectrum.

The lattice constant of the cubic structure of In_2_O_3_ was calculated using the following equation [13]:

(1) $$\begin{array}{c} d_{hkl}=\frac{a}{\sqrt{h^{2}+k^{2}+l^{2}}}, \end{array}$$

where *a* is the lattice constant and *d*_*hkl*_ is the interplanar spacing. The calculated values of *a* are listed in Table 1. The values of *a* decreased and are closer to the bulk one as the annealing temperature was increased from 350°C to 550°C. The large value of *a* denotes that the unit cell is elongated and compact pressure is in the plane of the film [14]. Yuan et al. mentioned that the increment in annealing temperature caused the compressive strain in the In_2_O_3_ film to be relaxed constantly [14]. The film annealed at 550°C is almost fully relaxed as the value of *a* was smaller than bulk crystal one.

Furthermore, the crystallite sizes of the cubic In_2_O_3_ can be calculated using Debye-Scherrer's formula [15]:

(2) $$\begin{array}{c} D=\frac{k\lambda }{\beta \cos\theta }, \end{array}$$

where *D* is the crystallite size, *k* is Scherrer's constant (*k* = 0.94), *λ* is X-ray wavelength with 0.15406 nm, *β* is the full width at half maximum (FWHM) of the Bragg peak, and *θ* is the Bragg angle. The crystallite size of the films increased as the annealing temperature was increased due to enhancement in the crystallinity of the films. The narrow diffraction of the film annealed at 450°C exhibited larger average crystallite sizes than the other annealed films.

Moreover, the number of defects in the thin film can be determined by calculating the dislocation density, *δ*, by using the following equation [16]:

(3) $$\begin{array}{c} \delta =\frac{1}{D^{2}}. \end{array}$$

The dislocation density decreased as the annealing temperature was increased as listed in Table 1. The dislocation density is very large at annealing temperature of 350°C. The annealing treatment with high electron mobility has decreased the amount of defects on the high-annealed film and thus changes the surface structures. The transformation in morphology of the films can be observed through the FESEM images.

### 3.2. Morphological Characterization

**Figure 3:**
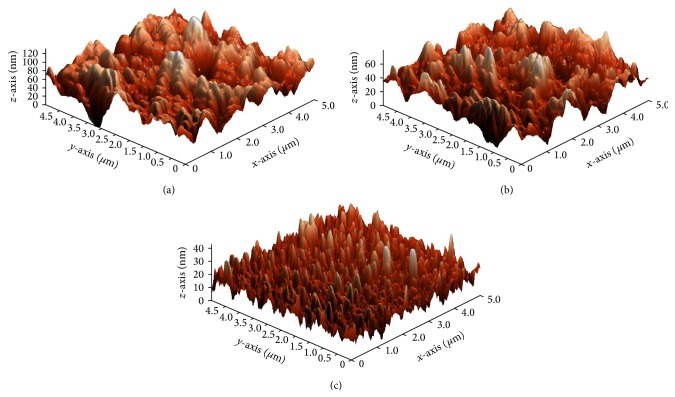
AFM images of In_2_O_3_ thin films annealed at (a) 350°C, (b) 450°C, and (c) 550°C.

AFM analysis shows clearly the morphology characterization to examine the difference in roughness between the thermally treated In_2_O_3_ thin films. The previous study reported that AFM is an efficient tool to study the surface morphology and microtribological properties of thin films [17, 18]. Figures 3(a), 3(b), and 3(c) show the three-dimensional surface morphology of the In_2_O_3_ thin films annealed at 350°C, 450°C, and 550°C, respectively.

The RMS values of the annealed thin films were tabulated in Table 2. In_2_O_3_ film annealed at 350°C showed around 2 nm of RMS surface roughness with granular structure (Figure 2(a)). The compact nature was seen in the films with high annealing temperature. Low annealed In_2_O_3_ thin film was identified as nonhomogeneous structure with a smooth surface area. In contrast, the high annealed films above 350°C displayed rougher structure with RMS value ranging around 11–29 nm (Figure 2(b)). The obtained AFM results were similar to the research of Beena et al. [19]. Hence, AFM analysis denoted that In_2_O_3_ thin film annealed at high annealing temperature showed more homogeneous structure with rougher surface roughness compared to low-annealed In_2_O_3_ films.

**Figure 4:**
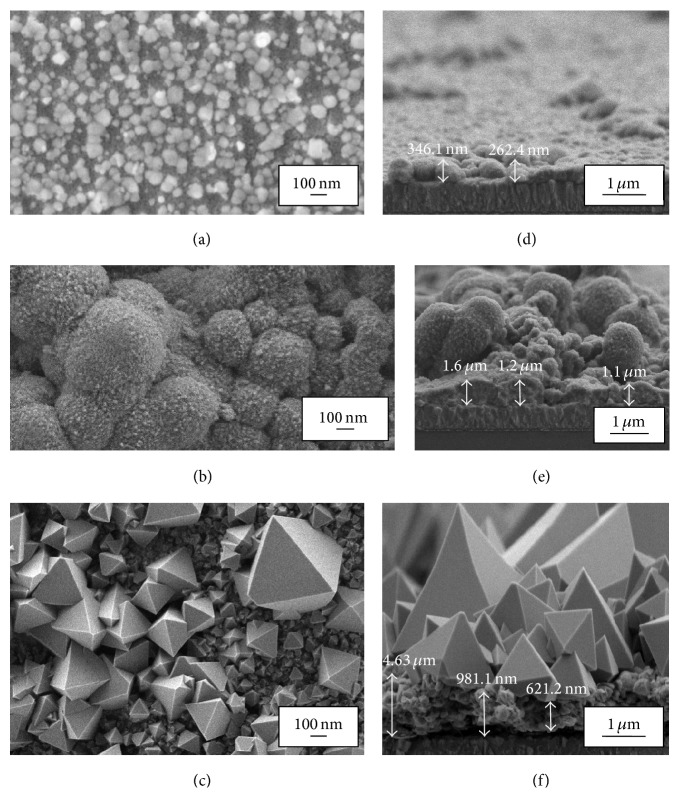
FESEM images of In_2_O_3_ thin films annealed at (a) 350°C, (b) 450°C, and (c) 550°C and thickness of In_2_O_3_ thin films annealed at (d) 350°C, (e) 450°C, and (f) 550°C.

Moreover, the FESEM (Figure 4) results showed roughness structures similar to the results showed in AFM analysis (Figure 3). The In_2_O_3_ thin film signifies very apparent smooth surface area or nonhomogeneous structures of In_2_O_3_ aggregations in Figure 4(a). In nanoscale (Figure 4(b)) the highly porous In_2_O_3_ structure has large nanoparticles in a diameter range of 121 nm. The sphere shape nanoparticles covered with tiny fur gives a direct pathway to the photogenerated electrons to be diffused easily into the working electrode. Comparatively, the pyramid-like octahedral structure observed in Figure 4(c) due to high-annealing temperature at 550°C shows rougher surface which tallies with the larger RMS value obtained in Table 2. The diameter of the octahedral In_2_O_3_ was measured to be 410 nm which is larger than the nanoparticles observed in Figure 4(b). The octahedral structure observed in the FESEM images is proved in the orientation of (111) crystal plane from the XRD spectrum (Figure 2).

**Figure 5:**
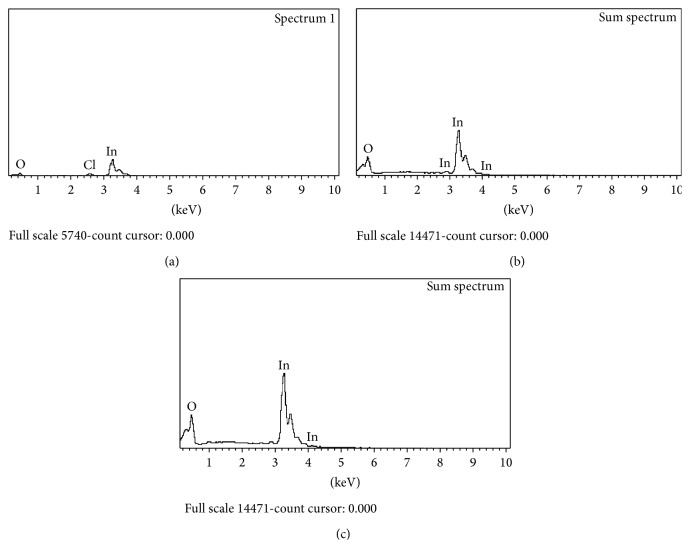
EDX Sampling of the In_2_O_3_ thin films annealed at (a) 350°C, (b) 450°C, and 550°C.

The thickness of the film is shown in Figure 4 for thin films annealed at (d) 350°C, (e) 450°C, and (f) 550°C. Thickness of the thin film increased as the annealing temperature increased as seen in Table 2. The EDX images are shown in Figure 5. The presence of chloride is obviously seen in the thin film annealed at 350°C which was also traced in the XRD peaks (Figure 2). The In_2_O_3_ thin films annealed at 450°C and 550°C show only indium (In) and oxygen (O) elements from the EDX spectrum. In addition, the EDX spectrum also analyzed the weight % of the oxygen compound which increased gradually as the annealing temperature increases (Table 2).

**Figure 6:**
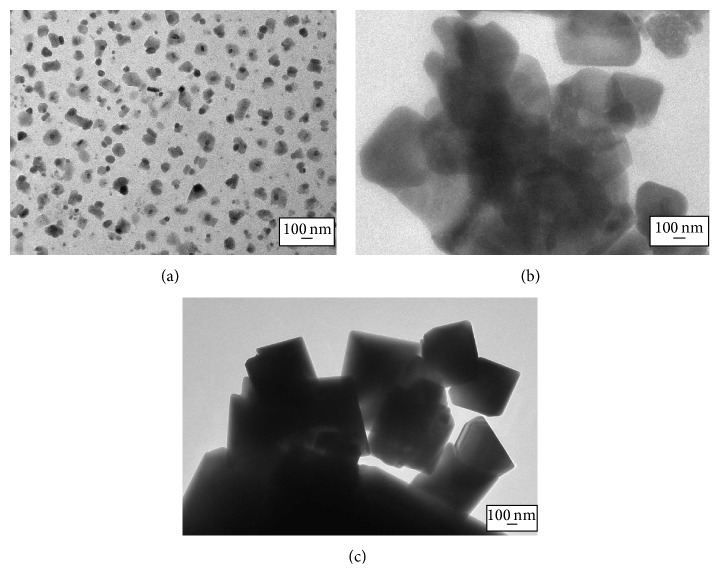
TEM images of In_2_O_3_ thin films annealed at (a) 350°C, (b) 450°C, and (c) 550°C.

Furthermore, TEM images in Figure 6 revealed the inner structures of the annealed In_2_O_3_ thin films. The particle size measured in the TEM images were more or less similar to the one measured in the FESEM images. From Table 2, the diameter of the In_2_O_3_ particles increased as the annealing temperature was increased.

### 3.3. Photovoltaic Performance of DSSC

**Figure 7:**
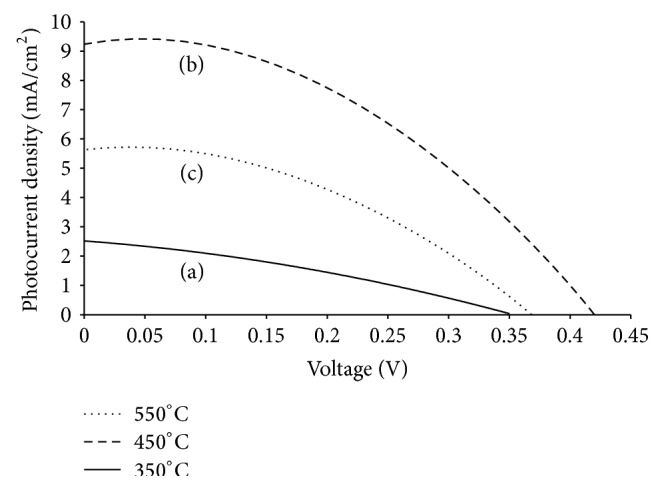
*J*-*V* characteristics of In_2_O_3_ thin films annealed at (a) 350°C, (b) 450°C, and (c) 550°C.

Figures 7(a), 7(b), and 7(c) showed the graphical image of the *J*-*V* characteristics and the corresponding photovoltaic parameters of In_2_O_3_ annealed at 350°C, 450°C, and 550°C, respectively.  Table 3 lists the corresponding photovoltaic properties. The results show an increment in *J*_sc_ and *V*_oc_ of DSSC as the annealing temperature increased from 350°C to 450°C. This increment is due to the higher porosity in the thin film that enables dye molecules to be absorbed easily into the thin film [20]. Besides that, the fill factor (FF) for film annealed at 450°C showed the highest value of 0.43. The film annealed at high temperature of 550°C has a lower *J*_sc_ and *V*_oc_ than film of 450°C but higher ones than film annealed at 350°C. This result indicates that heat treatment enhances the photovoltaic properties in DSSCs [21]. However, the photovoltaic efficiency of the film annealed at 550°C exhibits lower efficiency than film annealed at 450°C. This can be attributable to the greater thickness of the film where Hamadanian et al. mentioned that excess thickness in the photoanode layer decreases the performance of DSSCs because of electron recombination that takes place in the cell [22]. Therefore, the optimum annealing temperature for In_2_O_3_-based DSSCs is 450°C with power conversion efficiency (*η*) of 1.54%.

### 3.4. Electron Mobility

**Figure 8:**
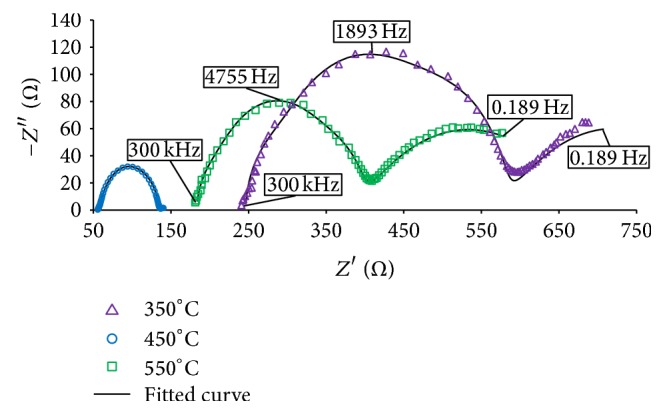
EIS spectra with fitted curves of the annealed In_2_O_3_ thin films.

**Figure 9:**
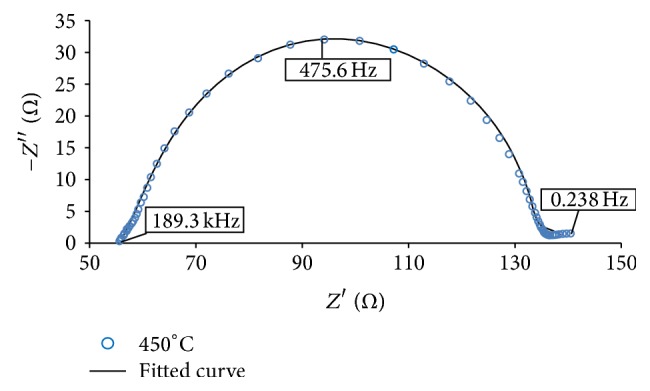
Magnified image of In_2_O_3_ thin film annealed at 450°C.

**Figure 10:**
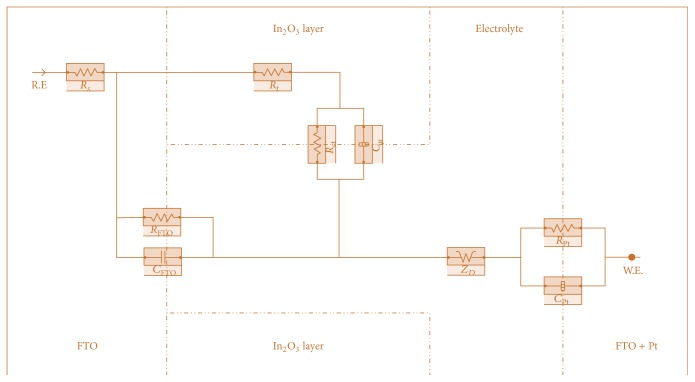
Equivalent circuit model to be fitted in EIS spectra.

Figure 8 shows the EIS spectra with fitted curves of the annealed thin films.  Figure 9 illustrates the magnified version of the film annealed at 450°C. The spectra were fitted based on a transmission line equivalent circuit proposed by early researchers [5, 23–25]. The circuit is shown in Figure 10 and the electron transport properties obtained from the fitted curves are listed in Table 4. The overall physical structure of the DSSC is illustrated in the model including the capacitances and resistances that exist in the cell [23]. The elements that exist in the cell are sheet resistance (*R*_*s*_), substrate resistance (*R*_FTO_), substrate capacitance (*C*_FTO_), transport resistance (*R*_*t*_), charge transfer resistance (*R*_ct_), chemical capacitance (*C*_*µ*_), Warburg impedance (*Z*_*D*_), counter electrode resistance (*R*_Pt_), and Helmholtz capacitance (*C*_Pt_) [23–25].

Moreover, the peak frequency of the In_2_O_3_ semicircles is *ω*_max_ or *f*_max_ (475.6–4755 Hz). *ω*_max_ can be presented as follows [26]:

(4) $$\begin{array}{c} \omega_{max}={\tau_{eff}}^{-1}, \end{array}$$

where *τ*_eff_ is the electron lifetime of the cell [27]:

(5) $$\begin{array}{c} \tau_{eff}=\frac{1}{2\pi f_{max}}. \end{array}$$

In addition, the thickness of the photoanode (*L*) and the electron diffusion length (*L*_*n*_) are related with *τ*_eff_ and the electron diffusion coefficient (*D*_eff_), where [26]

(6) Deff=RctL2Rtτeff,Ln=Deff×τeff.

The electron movement across the In_2_O_3_ layer (*ω*_*d*_) and frequency of the carrier transport process (*ω*_rec_) can be described as [24]

(7) $$\begin{array}{c} \frac{\omega_{d}}{\omega_{rec}}=\frac{R_{ct}}{R_{t}}={\left(\;\frac{L_{n}}{L}\;\right)}^{2}. \end{array}$$

From Table 4, In_2_O_3_-450°C-based DSSC obtained the longest *τ*_eff_ of 0.21 ms than the other annealed thin films. The highest *τ*_eff_ signifies low recombination rate between the excited electrons and the electrolyte layer [28]. In order to have good device performance, the electron diffusion length should be greater than the photoanode length (*L*_*n*_ ≫ *L*) and the charge transfer resistance should be larger than the transport resistance (*R*_ct_ ≫ *R*_*t*_) [29]. Greater *L*_*n*_ enables the dye molecules to harvest more incident photons [30].

Although the film annealed at high temperature of 550°C has a better crystalline properties as seen in the XRD spectrum (Figure 2), it showed poor electron transport properties. The same result was observed in Abdullah et al. where the thin films' crystalline properties increased as the annealing temperature increased but showed shorter electron diffusion length in the respective thin films [10, 31]. It is well known that annealing improves the crystalline properties of thin films by relaxing the compressive strains [14, 32]. However, it does not mean that thin films with greater crystalline properties possess good electron transport. The film annealed at 550°C obtained shorter *L*_*n*_ of 1.59 × 10^−6^ *µ*m which can be due to greater thickness of photoanode layer (*L*) of 1.4 *µ*m (Figure 4(f)). The excess thickness in the layer can inhibit the DSSC performance by increasing the electron recombination rate in the cell [22].

It was mentioned earlier that, for a desired case in DSSC, the length of excited electron should be the same as or more than the thickness of the photoanode (*L*_*n*_ ≫ *L*) which is actually to indicate that the electron had reached the working electrode successfully by diffusing through the In_2_O_3_ layer with a slow recombination process. In the film annealed at 550°C, it is proven that most of the photogenerated electrons failed to reach the working electrode due to strong recombination process occurring in the cell (*L*_*n*_ ≪ *L*). The strong electron recombination effect in film annealed at 550°C can be observed by analyzing the values of *R*_ct_ and *R*_*t*_. It was mentioned above that *R*_ct_ ≫ *R*_*t*_ for a good DSSC performance. It is clearly seen that a strong recombination effect takes place in the cell (*R*_ct_ ≪ *R*_*t*_) and eventually reduces the electron diffusion in the cell and causes small *D*_eff_ of 0.473 × 10^−3^ cm^2^/s. Bisquert and Fabregat-Santiago outlined *C*_*µ*_ related with photoanode Fermi level increment [33]. The film, annealed at 550°C, obtained 7.9 *µ*F. Therefore, low *R*_ct_ and *C*_*µ*_ seriously affected the power conversion efficiency of the cell to 0.73%. This type of undesirable case is called Gerishcher impedance [34].

In contrast, the In_2_O_3_-450°C caused most of the excited electrons to diffuse through the photoanode layer and reach the working electrode with slow recombination effect. The film obtained larger *D*_eff_ around 2.43 × 10^−3^ which provides high electron transport rate and thus improves *J*_sc_ and *V*_oc_ of the cell. Furthermore, the value of *R*_ct_ ≫ *R*_*t*_ supports the high efficiency in the film annealed at 450°C. The low recombination effect increased more electrons to diffuse into the photoanode. Lower resistance of charge transfer in the cell caused slow recombination of electron-hole and speeds up *τ*_eff_ [30].

Hence, the greater performance of the film annealed at 450°C has faster electron transport with low recombination rate and high diffusion rate. Besides that, annealing plays a major role in enhancing performance of DSSCs where an optimum annealing temperature needs to be known. In this work, the optimum annealing temperature for In_2_O_3_-based DSSC is 450°C. The EIS analysis showed a clear picture of the high and low performance of each annealed thin films.

## 4. Conclusion

In summary, we have successfully investigated the influence of annealing temperature in In_2_O_3_-based DSSCs. The films showed better crystallinity, rougher structure, and thicker photoanode layer as the annealing temperature increased from 350 to 550°C. The film annealed at 450°C exhibited highest power conversion efficiency of 1.54%. Although, the film annealed at 550°C has better crystalline properties, the greater thickness of the photoanode layer degraded the DSSC performance. Stronger recombination effect was observed in the films annealed at 350°C and 550°C where *R*_*t*_ ≫ *R*_ct_ with small *D*_eff_. The high recombination effect caused slower diffusion of injected electrons in the photoanode. Thus, *L* ≫ *L*_*n*_. In contrast, the film annealed at 450°C has larger *D*_eff_ (2.43 × 10^−3^) and small amount of charge transfer *R*_ct_ ≫ *R*_*t*_, thus increasing *τ*_eff_ (0.21 ms) of the cell. Therefore, the current findings greatly suggested that annealing temperature of 450°C acquires good structural, morphology, and electrical properties for efficient In_2_O_3_-based DSSCs.

## Figures and Tables

**Table 1:** XRD parameters of the In_2_O_3_ thin films annealed at 350°C, 450°C, and 550°C.

Annealing temperature (°C)	*hkl*	2*θ* (°)	*d*_*hkl*_ (nm)	*a* (nm)	*D* (nm)	*δ* (line^2^/m^2^)
350	(222)	30.525	0.296	10.136	8.3810	14.237 × 10^15^
450	(222)	30.550	0.292	10.129	17.188	3.385 × 10^15^
550	(222)	30.625	0.292	10.105	19.101	2.741 × 10^15^

**Table 2:** RMS surface roughness of the annealed thin films.

Annealing temperature (°C)	RMS (nm)	Thickness	Oxygen element (weight %)	Particle size (nm)	
				FESEM	TEM
350	2	346.1 nm	19.37	52	62
450	11	591.7 nm	22.37	121	169
550	29	1.4 *μ*m	27.67	410	478

**Table 3:** Photovoltaic parameters of In_2_O_3 _thin film annealed at 350°C, 450°C, and 550°C.

Annealing temperature (°C)	*V*_oc_ (V)	*J*_sc_ (mA/cm^2^)	FF	*η* (%)
350	0.35	2.5	0.32	0.28
450	0.42	9.2	0.43	1.54
550	0.36	5.8	0.35	0.73

**Table 4:** Electron transport properties obtained from EIS analysis.

Temperature (°C)	*L* (*μ*m)	*ω*_max⁡_ (Hz)	*R*_ct_ (Ω)	*R*_*t*_ (Ω)	*C*_*µ*_ (*μ*F)	*τ*_eff_ (ms)	*D*_eff_ (cm^2^s^−1^) ×10^−3^	*L*_*n*_ (*μ*m)	*η* (%)
350	0.35	1893	26.20	215	0.16	0.53	0.00028	0.12	0.28
450	1.60	476	641	3.21	6.01	2.10	2.43	22.6	1.54
550	4.63	4755	32.12	198	0.52	0.21	0.165	1.86	0.73

## References

[1] O'Regan B., Graetzel M. (1991). Low-cost, high-efficiency solar cell based on dye-sensitized colloidal TiO2 films. *Nature*.

[2] Abdullah H., Atiqah N. A., Omar A. (2015). Structural, morphological, electrical and electron transport studies in ZnO-rGO (wt% = 0.01, 0.05 and 0.1) based dye-sensitized solar cell. *Journal of Materials Science: Materials in Electronics*.

[3] An H., Xue B., Li D. (2006). Environmentally friendly LiI/ethanol based gel electrolyte for dye-sensitized solar cells. *Electrochemistry Communications*.

[4] Burschka J., Pellet N., Moon S.-J. (2013). Sequential deposition as a route to high-performance perovskite-sensitized solar cells. *Nature*.

[5] Shang G., Wu J., Tang S. (2012). Preparation of hierarchical tin oxide microspheres and their application in dye-sensitized solar cells. *Journal of Materials Chemistry*.

[6] Mahalingam S., Abdullah H., Omar A. (2015). Effect of morphology on SnO_2_/MWCNT-based DSSC performance with various annealing temperatures. *Advanced Materials Research*.

[7] Abdullah H., Mahalingam S., Omar A., Razali M. Z., Shaari S., Asshari I. (2014). Platinum-incorporating graphene counter electrode for In_2_O_3_-based DSSC with various annealing temperatures. *Advanced Materials Research*.

[8] Gan J., Lu X., Wu J. (2013). Oxygen vacancies promoting photoelectrochemical performance of In_2_O_3_ nanocubes. *Scientific Reports*.

[9] Sharma R., Mane R. S., Min S.-K., Han S.-H. (2009). Optimization of growth of In_2_O_3_ nano-spheres thin films by electrodeposition for dye-sensitized solar cells. *Journal of Alloys and Compounds*.

[10] Abdullah H., Razali M. Z., Shaari S., Taha M. R. (2014). Enhancement of dye-sensitized solar cell efficiency using carbon nanotube/TiO_2_ nanocomposite thin films fabricated at various annealing temperatures. *Electronic Materials Letters*.

[11] Jana A., Das P. P., Agarkar S. A., Sujatha Devi P. (2014). A comparative study on the dye sensitized solar cell performance of solution processed ZnO. *Solar Energy*.

[12] Chen Y., Zhou X., Zhao X., He X., Gu X. (2008). Crystallite structure, surface morphology and optical properties of In_2_O_3_-TiO_2_ composite thin films by sol-gel method. *Materials Science and Engineering: B*.

[13] Bucak S., Rende D. (2014). *Colloid and Surface Chemistry: A Laboratory Guide for Exploration of the Nano World*.

[14] Yuan Z., Zhu X., Wang X. (2011). Annealing effects of In_2_O_3_ thin films on electrical properties and application in thin film transistors. *Thin Solid Films*.

[15] Klug H. P., Alexander L. E. (1974). *X-Ray Diffraction Procedures*.

[16] El-Nahass M. M., Zeyada H. M., Aziz M. S., El-Ghamaz N. A. (2002). Optical properties of thermally evaporated SnS thin films. *Optical Materials*.

[17] Bhushan B., Israelachvili J. N., Landman U. (1995). Nanotribology: friction, wear and lubrication at the atomic scale. *Nature*.

[18] McKendry R., Theoclitou M.-E., Rayment T., Abell C. (1998). Chiral discrimination by chemical force microscopy. *Nature*.

[19] Beena D., Lethy K. J., Vinodkumar R., Detty A. P., Mahadevan Pillai V. P., Ganesan V. (2010). Photoluminescence in laser ablated nanostructured indium oxide thin films. *Journal of Alloys and Compounds*.

[20] Kao M. C. (2011). Effect of annealing temperature of TiO_2_ thin films for application in dye-sensitized solar cell. *Hsiuping Journal*.

[21] Li L., Xu C., Zhao Y., Ziegler K. J. (2014). Tin-doped indium oxide-titania core-shell nanostructures for dye-sensitized solar cells. *Advances in Condensed Matter Physics*.

[22] Hamadanian M., Gravand A., Farangi M., Jabbari V. The effect of the thickness of nanoporous TiO_2_ film on the nanocrystalline dye-sensitized solar cell.

[23] Zhang Y., Ram M. K., Stefanakos E. K., Goswami D. Y. (2012). Synthesis, characterization, and applications of ZnO nanowires. *Journal of Nanomaterials*.

[24] Bisquert J. (2002). Theory of the impedance of electron diffusion and recombination in a thin layer. *Journal of Physical Chemistry B*.

[25] Fabregat-Santiago F., Bisquert J., Garcia-Belmonte G., Boschloo G., Hagfeldt A. (2005). Influence of electrolyte in transport and recombination in dye-sensitized solar cells studied by impedance spectroscopy. *Solar Energy Materials and Solar Cells*.

[26] Adachi M., Sakamoto M., Jiu J., Ogata Y., Isoda S. (2006). Determination of parameters of electron transport in dye-sensitized solar cells using electrochemical impedance spectroscopy. *Journal of Physical Chemistry B*.

[27] Wang Q., Ito S., Grätzel M. (2006). Characteristics of high efficiency dye-sensitized solar cells. *Journal of Physical Chemistry B*.

[28] Wang Q., Moser J., Grätzel M. (2005). Electrochemical impedance spectroscopic analysis of dye-sensitized solar cells. *The Journal of Physical Chemistry B*.

[29] Bisquert J., Fabregat-Santiago F., Mora-Seró I., Garcia-Belmonte G., Giménez S. (2009). Electron lifetime in dye-sensitized solar cells: theory and interpretation of measurements. *Journal of Physical Chemistry C*.

[30] Martinson A. B. F., McGarrah J. E., Parpia M. O. K., Hupp J. T. (2006). Dynamics of charge transport and recombination in ZnO nanorod array dye-sensitized solar cells. *Physical Chemistry Chemical Physics*.

[31] Abdullah H., Omar A., Asshaari I. (2015). *Advanced Processing and Manufacturing Technologies for Nanostructured and Multifunctional Materials*.

[32] Chung J., Lee J., Lim S. (2010). Annealing effects of ZnO nanorods on dye-sensitized solar cell efficiency. *Physica B: Condensed Matter*.

[33] Bisquert J., Fabregat-Santiago F., Kalyanasundaram K. (2010). Impedance spectroscopy: a general introduction and application to dye-sensitized solar cells. *Dye-Sensitized Solar Cells*.

[34] Andersen A. R., Halme J., Lund T. (2011). Charge transport and photocurrent generation characteristics in dye solar cells containing thermally degraded N719 dye molecules. *The Journal of Physical Chemistry C*.

